# Diagnostic values of ultrasound and the Modified Alvarado Scoring System in acute appendicitis

**DOI:** 10.1186/1865-1380-5-26

**Published:** 2012-06-06

**Authors:** Shirzad Nasiri, Fatemeh Mohebbi, Nassim Sodagari, Anushiravan Hedayat

**Affiliations:** 1 Tehran University of Medical Sciences, Shariati Hospital, North Karegar Avenue, Tehran, Iran; 2 Resident of General Surgery, Shariati Hospital, Tehran University of Medical Sciences, Tehran, Iran

**Keywords:** Appendicitis, Ultrasonography, Modified Alvarado Scoring System (MASS)

## Abstract

**Background:**

Making the diagnosis of acute appendicitis is difficult, and is important for preventing perforation of the appendix and negative appendectomy results. Ultrasound and clinical scoring systems are very helpful in making the diagnosis. Ultrasound is non-invasive, available and cost-effective, and can accomplish more than CT scans. However, there is no certainty about its effect on the clinical outcomes of patients, and it is operator dependent. Counting the neutrophils as a parameter of the Alvarado Scale is not routine in many laboratories, so we decided to evaluate the diagnostic value of the Modified Alvarado Scaling System (MASS) by omitting the neutrophil count and ultrasonography.

**Methods:**

After ethical approval of methodology in Tehran University of Medical Sciences ethical committee, we collected the data. During 9 months, 75 patients with right lower quadrant pain were enrolled in the study, and underwent abdominal ultrasonography and appendectomy, with pathological evaluation of the appendix. The MASS score was calculated for these patients and compared with pathology results.

**Results:**

Fifty-five male and 20 female patients were assessed. Of these patients 89.3% had acute appendicitis. The sensitivity, specificity, PPV, NPV and accuracy rate of ultrasonography was 71.2%, 83.3%, 97.4%, 25% and 72.4%, respectively. By taking a cutoff point of 7 for the MASS score, a sensitivity of 65.7%, specificity of 37.5%, PPV of 89.8%, NPV of 11.5% and accuracy of 62.7% were calculated. Using the cutoff point of 6, a sensitivity of 85.1%, specificity of 25%, PPV of 90.5%, NPV of 16.7% and accuracy of 78.7% were obtained.

**Conclusion:**

Ultrasound provides reliable findings for helping to diagnose acute appendicitis in our hospital. A cutoff point of 6 for the MASS score will yield more sensitivity and a better diagnosis of appendicitis, though with an increase in negative appendectomy.

## Background

Acute appendicitis is one of the most common and challenging surgical emergencies, and can lead to appendiceal perforation and peritonitis, which are concomitant with high mortality and morbidity [1]. Making the decision for a surgical operation based only on the patient’s signs and symptoms results in removing normal appendices (negative appendectomy) in 15% to 30% of cases. [2-4] The rational approach is to decrease the negative appendectomy as well as appendiceal rupture rates. A decrease in unnecessary appendectomies should not cause an increase in perforation rates [5,6].

For this reason, a number of diagnostic modalities have been proposed, including laparoscopy, clinical scoring systems, computer programs, ultrasonography, CT scans and MRI [7-9]. Imaging techniques are fairly accurate [10,11]. Graded compression ultrasonography is an inexpensive, fast and noninvasive method with an accuracy rate of 71%–90% for the diagnosis of acute appendicitis [12-14], but there is no certainty about the effect of ultrasonography on the clinical outcomes of patients [13,15]. Furthermore, clinical judgment should not be abandoned because of the lack of ultrasound findings in patients with a high probability of acute appendicitis [16]. Also, ultrasonography is an operator-dependent modality, and the diagnostic values are different in various studies. [2,17-19]

The likelihood of appendicitis is ascertained by the Alvarado Scoring System [20]. It is accepted that according to the Alvarado Scoring System, which consists of right lower quadrant tenderness, rebound tenderness, migrating pain, nausea and/or vomiting, anorexia, fever leukocytosis and a left shift in the leukocyte count [14,20], patients who get a score of 7 to 10 should undergo appendectomy, and patients with a score of 5 or 6 are candidates for a CT scan for the diagnosis [14]. Taking into consideration that counting the white blood cell (WBC) differentials is not routine in many laboratories, the Modified Alvarado Scoring System (MASS) was developed by omitting the left shift of leukocytosis from the Alvarado Scale [21].

Most hospitals in Iran do not count the neutrophils, and also the CT scans are not available. Therefore, we decided to evaluate the diagnostic value of the Modified Alvarado Scoring System (MASS) and the accuracy of graded compression ultrasonography in our setting (Shariati Hospital, one of the most important university hospitals in Tehran) for the diagnosis of acute appendicitis, comparing it with the gold standard of eventual pathology in order to obtain a cutoff point for the MASS score and also to assess the sensitivity and specificity of ultrasonography in our hospital.

## Methods

Over a 9-month period (December 2010-August 2011), a total of 75 patients were enrolled in our prospective study. Every patient who had come as a surgical emergency with right lower quadrant pain and underwent appendectomy was enrolled. Pathology reports of appendices were assessed. Exclusion criteria were appendiceal abscesses, phlegmon, evidence of generalized peritonitis and a palpable abdominal mass in the examination.

Ultrasound was carried out by radiology residents, and a noncompressible blind loop equal to or greater than 6 mm in anteroposterior diameter indicated appendicitis. The sensitivity and specificity of all ultrasonography images were calculated based on the pathology results of the appendectomy. Performing an ultrasound examination depended on the decision of the surgical team, and our study played no role in the management of the patients.

The Modified Alvarado Scoring System (MASS) criteria were fulfilled for each patient, and according to the pathology report, 75 patients (55 male, 20 female) were registered. MASS components were migration of pain, anorexia, nausea and/or vomiting, right lower quadrant tenderness, rebound tenderness, an elevated temperature of 37.5 ^0^ C or more, and leukocytosis (>10,000 WBCs). Right lower quadrant tenderness and leukocytosis had two scores, and the others had one score (Table 1).

**Table 1:** Modified Alvarado Scale

	***Manifestations***	***Value***
	Migration of pain	1
***Symptoms***	Anorexia	1
	Nausea and/or vomiting	1
***Signs***	Right lower quadrant tenderness	2
	Rebound	1
	Elevated temperature	1
***Laboratory values***	Leukocytosis	2
		Total points 9

Data were assessed by SPSS version 16, and a *p* value <0.05 was considered significant.

## Results and discussion

### Demographic results

Fifty-five males and 20 females were assessed. The mean age of the patients was 27 years (9 to 84 years old). Although the average age seemed to be higher in the female group (30 years in comparison with 25.9 years), the difference was not significant (*p* value > 0.05).

### Pathology results

Acute appendicitis was confirmed in 67 (89.3%) of the patients, and the remaining 8 (10.7%) patients had undergone negative appendectomies (Table 2).

**Table 2:** Pathology results

	***Female***	***Male***	
***Appendicitis***	16	51	67 (89.3%)
***Normal appendix***	4	4	8 (10.7%)

### Ultrasound results

Ultrasonography was performed on 39 male patients (71% of male patients) and 19 females (95% of female patients). Seventeen patients (16 men and 1 woman) without ultrasonography underwent appendectomies. Performance of ultrasonography was significantly higher in women (*p* value 0.002).

The positive predictive value (PPV) for ultrasonography was 97.4%, and the negative predictive value (NPV) was 25% in our study. The sensitivity for diagnosing acute appendicitis by ultrasound was 71.2%, the specificity was 83.3%, and the accuracy rate was 72.4% (Table 3).

**Table 3:** Ultrasonographic data

	***Appendicitis in ultrasonography***	***Normal ultrasonography***	***Total***
***Appendicitis***	37	15	52
***Normal appendix***	1	5	6
***Total***	38	20	58

### Modified Alvarado score system results (MASS)

Among the MASS components, right lower quadrant tenderness was the most common, and nausea and/or vomiting was significantly related with acute appendicitis (*p* value 0.001).

**Figure 1:**
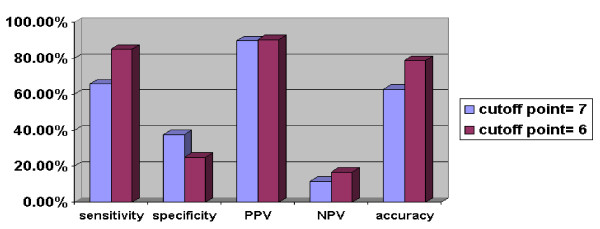
Modified Alvarado Scoring System with cutoff points of 6 and 7.

Figure 1 shows that 49 patients had MASS scores ≥ 7 and 26 patients had MASS scores < 7. Of these patients with a MASS score ≥ 7, five had a normal appendix according to pathology (negative appendectomy). Twenty-three patients with MASS scores < 7 had true appendicitis according to pathology. Therefore, the sensitivity was 65.7%, specificity 37.5%, PPV 89.8%, NPV 11.5% and accuracy 62.7% for the Modified Alvarado Scale with a cutoff point of 7.

**Figure 2:**
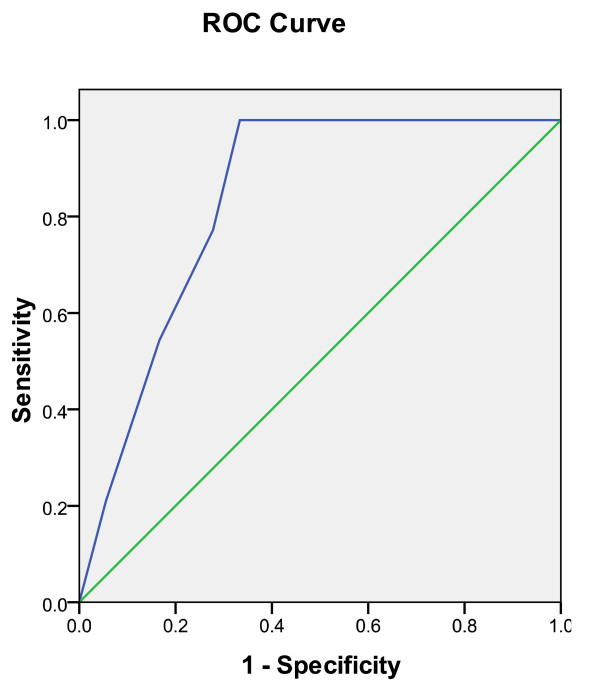
**ROC curve for diagnosis of acute appendicitis according to the Modified Alvarado Scoring System.** The area under the curve is 0.837 with a standard error of 0.67 and confidence interval of 0.705 to 0.968.

According to a cutoff point of 6, 63 patients had scores ≥ 6, and 12 patients had scores < 6. Six patients had negative pathology for appendicitis (negative appendectomy) and ten with a score < 6 had appendicitis according to the pathology report (false negative). Thus, the sensitivity of the MASS with a cutoff point of 6 was 85.1%, the specificity 25%, PPV 90.5% and NPV 16.7%, and the accuracy rate was 78.8% (Figure 2).

Decision-making in patients suspected of having acute appendicitis is still a diagnostic challenge worldwide despite the advances in appendiceal surgery and the decrease in mortality because of appendicitis [22]. According to some articles, negative appendectomy has been reported in 15% to 30% of appendectomies because of difficulties in making the diagnosis [4,23]. This can impose a significance burden on the health system. For instance, 39,901 patients underwent negative appendectomies in the US in 1997, which resulted in an estimated total hospital charge of 741.5 million dollars [24]. To assist and improve the diagnosis of acute appendicitis, a number of diagnostic modalities have been proposed, such as graded compression sonography and scoring systems [14].

Ultrasonography is an affordable, noninvasive tool whose result can be obtained more quickly than for CT scans [19]. Ultrasound has already been proved to have a high sensitivity and specificity in the diagnosis of acute appendicitis. Many data about are available, and 55% to 98% sensitivity and 78% to 100% specificity have been reported for ultrasonography [19,21]. Variations in reported data may be due to differences in study design, sample size, physician experience or applied statistical techniques of various studies. Ultrasound is an operator-dependent technique, and the results vary depending on who is performing the ultrasonography.

In our study, ultrasound had 71.2% sensitivity, 83.3%specificity and 72.4% accuracy. Comparing this study with others reveals that ultrasound provides reliable findings for the diagnosis of acute appendicitis in Shariati Hospital, even though it is done by radiology residents without much experience. The PPV of ultrasonography was 97.4%, and the NPV was 25%. These results emphasize again that a positive ultrasonography for appendicitis is strongly in favor of a diagnosis of acute appendicitis. However, a negative ultrasound is not sufficient to rule out the diagnosis and discharge the patient.

Ultrasound was performed significantly more often in women: 39 (71%) males and 19 (95%) females. This may indicate that equivocal cases of appendicitis that require diagnostic aids and modalities are more frequent in females.

The Alvarado Scoring System is based on signs, symptoms and laboratory data. It is a very sensitive tool for classifying patients with suspected acute appendicitis [20,23]. Taking into consideration that WBC differential counting is not easily and routinely done in many laboratories, the Modified Alvarado Scoring System (MASS), omitting the neutrophil count, has been used as an alternative. The MASS has been shown to be a quick and inexpensive diagnostic tool in patients suspected of suffering acute appendicitis. However, different accuracies have been reported for the MASS in different studies [14,21,24]. We found that the most common MASS parameter was right lower quadrant tenderness (85.3%), and the only factor whose correlation with acute appendicitis was statistically significant was nausea and/or vomiting. This could be due to the small sample size of our study concerning the detection of other significant correlations.

In his original article, Alvarado suggested that patients with scores of 7 or higher should be operated on [20]. In the same manner, for the MASS, the cutoff point of 7 has been commonly used [14,21,24]. In our investigation, a sensitivity of 65.7%, specificity of 37.5%, PPV of 89.8%, NPV of 11.5% and accuracy of 62.7% were obtained for a cutoff point of 7. In 2008, Sun et al. suggested that a cutoff point of 6 provides a higher sensitivity and NPV in the Alvarado system, and may be more appropriate in comparison with the traditional cutoff point of 7 [25].

Choosing the cutoff point of 6 in our study, the sensitivity of the MASS was 85.1%, specificity of 25%, PPV 90.5% and NPV 16.7%; the accuracy rate was 78.7%. Regarding these findings, it appears that a cutoff point of 6 for the MASS could be appropriate.

It would be more precise if we could include all patients suspected of having acute appendicitis and follow up those patients who did not undergo surgery, but patient follow-up has its own limitations, and an optimum follow-up was not possible for us. Moreover, we intended to have the pathology result of the resected appendix for the definite diagnosis. The estimated rate of negative appendectomy in our study was 10.7%, which is less than the accepted rate worldwide. We cannot make judgments about this rate until we have studied the perforation rate. In addition, a larger sample size is needed to estimate the precise negative appendectomy rate.

## Conclusion

Ultrasonography and Modified Alvarado Score are both beneficial in diagnosis of acute appendicitis. Though Ultrasonography is operator dependent, it has reasonable sensitivity and specificity in diagnosis. Moreover; a cutoff point of 6 for the MASS score will yield more sensitivity and a better diagnosis of appendicitis, though with an increase in negative appendectomy.

## Competing interests

The authors declare that they have no competing interests.

## Authors’ contributions

F.M participated in sequence alignment and study design. N.S helped to draft the manuscript and data analysis. A.H participated in revising and data interpretation. All authors read and approved the final manuscript.
